# Overexpression of plum auxin receptor *PslTIR1* in tomato alters plant growth, fruit development and fruit shelf-life characteristics

**DOI:** 10.1186/s12870-016-0746-z

**Published:** 2016-02-29

**Authors:** I. El-Sharkawy, S. Sherif, W. El Kayal, B. Jones, Z. Li, A. J. Sullivan, Subramanian Jayasankar

**Affiliations:** Department of Plant Agriculture, University of Guelph, Vineland Station, ON Canada; Damanhour University, Faculty of Agriculture, Damanhour, Egypt; The University of Sydney, Faculty of Agriculture, Sydney, Australia; Chongqing University, Genetic Engineering Research Center, Bioengineering College, Chongqing, China; Department of Plant Agriculture, University of Guelph, Guelph, ON Canada

**Keywords:** Auxin receptors, Auxin/ethylene cross-talk, Cell-wall metabolism, Fruit-set, Fruit firmness, Plant development, Reproductive growth, Shelf-life

## Abstract

**Background:**

TIR1-like proteins are *F*-box auxin receptors. Auxin binding to the *F*-box receptor proteins promotes the formation of SCF^TIR1^ ubiquitin ligase complex that targets the auxin repressors, Aux/IAAs, for degradation via the ubiquitin/26S proteasome pathway. The release of auxin response factors (ARFs) from their Aux/IAA partners allows ARFs to mediate auxin-responsive changes in downstream gene transcription. In an attempt to understand the potential role of auxin during fruit development, a plum auxin receptor, *PslTIR1,* has previously been characterized at the cellular, biochemical and molecular levels, but the biological significance of this protein is still lacking. In the present study, tomato (*Solanum lycopersicum)* was used as a model to investigate the phenotypic and molecular changes associated with the overexpression of *PslTIR1*.

**Results:**

The findings of the present study highlighted the critical role of *PslTIR1* as positive regulator of auxin-signalling in coordinating the development of leaves and fruits. This was manifested by the entire leaf morphology of transgenic tomato plants compared to the wild-type compound leaf patterning. Moreover, transgenic plants produced parthenocarpic fruits, a characteristic property of auxin hypersensitivity. The autocatalytic ethylene production associated with the ripening of climacteric fruits was not significantly altered in transgenic tomato fruits. Nevertheless, the fruit shelf-life characteristics were affected by transgene presence, mainly through enhancing fruit softening rate. The short shelf-life of transgenic tomatoes was associated with dramatic upregulation of several genes encoding proteins involved in cell-wall degradation, which determine fruit softening and subsequent fruit shelf-life.

**Conclusions:**

The present study sheds light into the involvement of *PslTIR1* in regulating leaf morphology, fruit development and fruit softening-associated ripening, but not autocatalytic ethylene production. The results demonstrate that auxin accelerates fruit softening independently of ethylene action and this is probably mediated through the upregulation of many cell-wall metabolism genes.

**Electronic supplementary material:**

The online version of this article (doi:10.1186/s12870-016-0746-z) contains supplementary material, which is available to authorized users.

## Background

The phytohormone auxin controls almost every aspect of plant growth and development. At cellular level, auxin regulates cell division, expansion and differentiation [1]. Some short-term effects may reflect direct auxin impact on cell membrane proteins; however, most other responses appear to be due to changes in the transcription of target auxin-responsive genes either by activation or repression [2, 3]. At the whole plant level, auxin controls essential processes such as apical dominance, lateral root formation, tropic responses, vascular initiation and differentiation, embryogenesis, and fruit development [4, 5].

Fruit development is a multiphase process that requires a tight coordination among molecular, biochemical and structural elements. The series of modifications that make the fruit proceed through consequent developmental stages involve many distinctive metabolic pathways. The availability of plant mutants with fruits unable to ripen autonomously has helped us to understand the mechanisms underlying fruit development process in which phytohormones are placed as master regulators, leading to efficient reproductive growth [6, 7]. Among various phytohormones, auxin has received a lot of attention due to its prominent role in controlling wide-range of events during plant life, particularly those involved in flower and fruit development [1, 8]. In flowering plants, auxin is required for floral meristem formation and acts with homeotic genes in determining floral organogenesis [9]. Auxin bioassays highlight the pivotal role played by auxin in regulating the reproductive growth and the final fruit size through coordinating the abundant cell division and expansion that occurs after anthesis [10–13]. The transition of ovary into fruit is initiated by successful pollination and fertilization [11], in which auxin plays key role in triggering the fruit-set program and initiating fruit development [14–16]. Further, several reports demonstrated the critical role played by auxin in regulating the onset and coordination of ripening processes, and subsequent fruit shelf-life [6, 11, 13, 17–19]. Recent studies have shown that auxin accelerates fruit development and ethylene production, acting at least partially by triggering the expression of several ethylene biosynthesis and response components [13, 20, 21]. Once the ripening process is initiated, it cannot be stalled and generally leads to over-ripening that in turn negatively affects fruit quality. Therefore, identifying factors that coordinate fruit ripening remains one of the biggest challenges to minimize postharvest losses. Several fruit parameters are used to specify the progression of ripening. The significant postharvest loss of fresh fruits due to excessive and rapid softening has urged considerable research into investigating the mechanisms that underlie cell-wall dynamics [19, 22, 23]. Fruit textural changes during ripening are associated with numerous modifications of the cell-wall architecture, leading to a reduction in intercellular adhesion, depolymerization and solubilization of pectins and hemicellulose, and loss of pectic galactose side chains [24, 25]. These modifications in cell-walls involve the coordinated and interdependent action of many cell-wall modifying enzymes and proteins. Thus, investigating the developmental process and signal mechanisms involved in the regulation of cell-wall associated genes is an important area of research. Despite the well-established role played by ethylene in orchestrating the ripening of climacteric fruit, the role of other ethylene-independent metabolic pathways in the regulation of climacteric fruit ripening is obvious [6, 26, 27]. Indeed, several studies have accentuated the impact of auxin on regulating different aspects of fruit ripening and quality traits in many crop species [19, 28–30]. For instance, manipulation of auxin-signalling components in tomato can enhance starch accumulation, increase wall thickness of fruit epidermal cells, and reduce pectin solubilization [31–34]. However, the exact molecular mechanisms by which auxin regulates these processes are not fully understood.

It is well-established that auxin modulates plant development through transcriptional regulation of target auxin-responsive genes [3]. Therefore, the involvement of auxin in a diverse array of physiological functions should be equally mediated by a series of signalling network cascades. The discovery of the *F*-box proteins that act as auxin receptors has considerably improved our understanding of how auxin mediates cellular responses [35, 36]. Basically, the transcriptional regulators of Aux/IAAs and ARFs interact in homo- and heterodimers to form complexes that repress auxin-signalling. Auxin binding to the *F*-box receptor proteins promotes the formation of SCF^TIR1/AFB^ ubiquitin–ligase complexes that target the Aux/IAA repressors for degradation. This auxin-dependent proteolysis releases auxin response factors (ARFs) that otherwise remain trapped via their binding to Aux/IAA partners [37–40]. Loss of Aux/IAA allows ARF-mediated auxin-responsive changes in downstream gene transcription. Previous studies on tomato and *Arabidopsis* have identified several auxin-signalling components that act as positive or negative regulators of auxin responses, including a member of *F*-box auxin receptor (*TIR1*), an Aux/IAA transcription factor (*IAA9*), and two members of auxin-response factor, *ARF7* and *ARF8* [14, 16, 41–43]. The alteration of these auxin-signalling components either by activation (e.g. *TIR1*) or suppression (e.g. *IAA9*, *ARF7*, and *ARF8*) causes separation of fruit initiation from pollination and fertilization.

The role of auxin during the development of plum fruits has previously been demonstrated [17–19]. It was shown that exogenous application of auxin to plum fruits is capable of accelerating fruit development and ripening, confirming the role of auxin during ripening. However, the molecular mechanisms underlying such responses are still lacking. In the present study, a plum auxin receptor gene, *PslTIR1,* was overexpressed in tomato (*Solanum lycopersicum*), the plant model that has extensively been used to study ripening and postharvest biology of fleshy climacteric fruits. The morphological and molecular analysis of tomato transgenic lines clearly supported the hypothesis that auxin regulates leaf morphology, fruit development, and ripening through positive regulatory mechanisms. This study not only provide better understanding to the role of auxin-signalling components during fruit ripening, but might also lead to novel strategies for effective manipulation of ripening and fruit quality traits, adding a new level of complexity to the regulation of fruit ripening.

## Methods

### Plant materials and postharvest treatments

Tomato plants (*Solanum lycopersicum* cv. *Ailsa Craig*) were grown under controlled conditions set as follows: 14:10 h light/300 *μ*mol m^−2^ s^−1^; 25:20 °C and 80 % relative humidity. For molecular analysis, leaf samples were collected from 10 week old wild-type (WT) and T3 generation transgenic tomato lines. To evaluate the effect of auxin in the accumulation of early auxin-responsive genes, 12 d old WT tomato seedlings were soaked in liquid MS medium with or without (mock treatment) 10 μM NAA for 2 h. For ethylene quantification, WT and transgenic tomato fruit were harvested at early immature green, immature green, mature green, breaker, orange, early red, red, red-ripe stages. Ethylene production was quantified in 5 fruit/treatment/replicate with three independent biological replicates using gas chromatography. All fruit samples were frozen in liquid-N_2_ and stored at –80 °C for further analysis.

### Generation of transgenic tomato plants

A high fidelity PCR system was used to amplify the full-length sequence of *PslTIR1* cDNA [18], using gene specific primers 1 and 2 (Additional file 1: Table S1). The cDNA was fused into *Spe*I/*Bst*EII site of modified pCambia1304 binary vector (hygromycin resistant gene was replaced by kanamycin resistant gene) under the transcriptional control of *35S* promoter. The *PslTIR1* was then introduced into the WT tomato plants (*Solanum lycopersicum* cv. *Ailsa Craig*) by *Agrobacterium tumefaciens*-mediated transformation [32]. Transformed lines were first selected on kanamycin (70 mg L^−1^) and further confirmed by PCR with the genomic DNA (*g*DNA) extracted from leaves of 10 week old transgenic tomato lines to check the presence of T-DNA insertion. To identify discrete transgenic lines, a qPCR analysis was performed to determine *PslTIR1* transgene accumulation levels. Consequently, a number of independent transformation events were identified from which only two lines were selected for further analysis (L12 and L17). To determine parthenocarpic capacity, few flower buds of WT and transgenic plants were emasculated 2 d before anthesis to prevent self-pollination and all other flowers were removed.

### Nucleic acid extraction and qPCR assays

Total RNA extraction, DNase treatment, cDNA synthesis and qPCR reactions were performed as described previously [44]. Gene-specific primers were designed using Primer Express (v3.0, Applied Biosystems, Carlsbad, CA, USA) (Additional file 1: Table S1). Three technical replicates from three biological replicates for each reaction were analyzed on an ABI PRISM 7900HT Sequence Detection System (Applied Biosystems). Transcript abundance was quantified using standard curves for both target genes and tomato β-actin *SlAct* (BT013524) as a reference gene, which were generated from serial dilutions of PCR products from corresponding cDNAs. The expression level of *SlAct* among different tissues and treatments used in this study was assessed using absolute qPCR. The qPCR assay was performed based on the standard curve generated from recombinant plasmids. No significant differences in *SlAct* expression were detected between different treatments and tissue samples. We thus conclude that *SlAct* could be used as a reliable internal reference gene for qPCR. Genomic DNA was extracted from tomato leaves according to the DNeasy Plant Mini Kit (Qiagen, Mississauga, ON, Canada).

### Post-harvest treatments and shelf-life analysis

To evaluate the effects of auxin in fruit ripening and shelf-life characteristics, WT and transgenic tomato fruit were harvested at early breaker stage (~42 d after anthesis), surface sterilized, and subjected to various treatments (5 fruit/treatment/replicate; three biological replicates), including: 1-naphthalene acetic acid (R + N); NAA (10 μM/2 h), propylene (R + E); C_3_H_6_ (1000 μL L^−1^/24 h at 20 °C), the ethylene-inhibitor 1-methylcyclopropene (R + M); 1-MCP (1 μL L^−1^/24 h at 20 °C); and 1-MCP followed by dipping in NAA (R + M + N). Water-dipped fruit were used as controls (R). All fruit were kept at 20 °C until reaching red stage. In case of treatments with no obvious progression in ripening such as 1-MCP and 1-MCP/NAA treated fruit, samples were collected ~20 d after treatment. Fruit characteristics were assessed and sampled every 5 d until they lost their texture and structural integrity. To determine fruit physical properties as skin puncture strength, flesh firmness and weight loss, fruit were assessed at 0, 5, 10, 15 and 20 d of shelf-life. Loss of fruit weight was calculated as % of the initial fruit weight at harvest. Fruit firmness was measured in fruit with and without removal of skin to determine the skin punctures strength and the flesh compression mass using digital penetrometer equipped with a 3 mm cylinder probe (FHT200, Extech Instruments, USA). Each fruit was tested three times at equidistant points along the equatorial plane of the fruit.

### Statistical analysis

The significance of differences in expression data was tested on raw data by analysis of variance adopting the General Linear Model (GLM) using SAS software. Significance between mean values was estimated by Tukey’s HSD test carried out on raw data.

### Hierarchical clustering analysis

Gene expression of cell-wall metabolism genes that showed significant differences between WT and *PslTIR1* fruits and after exposure to different treatments that can alter auxin and ethylene signalling was grouped through a two-way hierarchical clustering. Pearson’s distance and Ward’s algorithm were used for data aggregation.

## Results and discussion

Despite the strong sequence structure conservation among all *TIR1/AFB* auxin receptors that support this common auxin-signalling mechanism [45], a number of studies have shown that TIR1/AFB proteins have distinct biochemical properties and biological functions. For example, the different TIR1/AFB proteins exhibited clear divergence in their binding properties for various auxin analogs, which consequently affect their auxin-dependent ability to assemble co-receptor complex with the different Aux/IAA proteins [46, 47]. Even in the presence of auxin, TIR1/AFBs demonstrated diverse capacities of assembling co-receptor pairs with Aux/IAAs, in which certain Aux/IAA proteins are generally better substrates than others for a specific TIR1/AFB protein. Accordingly, *Arabidopsis AFB3* has been shown to have a unique role in the nitrate response of roots [48]. Also, Tomato SlTIR1 and SlAFB6 have been shown to be involved in the auxin-signalling network controlling simplified leaf architecture formation [49]. Another dramatic impact that can discriminate between auxin receptors is the mechanism by how these receptors mediate auxin responses. Genetic studies indicated that the AFB4-class of auxin receptors negatively regulates the auxin-response, unlike other members of the family that act as positive regulators [47, 50, 51]. To add more level of complexity, classification of *F*-box auxin receptors gene family divided the different land plants TIR1/AFB members into four distinguishable clades on the basis of sequence structure; *TIR1*, *AFB2* (*AFB2/AFB3*), *AFB4* (*AFB4/AFB5*), and *AFB6* [27, 44]. It is worth noting that five and three *F*-box receptor members were identified in *Arabidopsis* and tomato, respectively. Despite that *AFB6* and *AFB2* homologs are absent in *Arabidopsis* and tomato, respectively; the consequences of their loss remained unclear. These observations suggest that the different TIR1/AFB gene members exhibit specific and overlapping biological functions. Therefore, determining the autonomous role of the different auxin receptors in plant development is necessary to understand the fundamental contribution of each protein in auxin-dependent plant responses. Recently, three plum genes encoding proteins closely related to the *TIR1*-like gene family of auxin receptors (*PslTIR1*, *PslAFB2*, and *PslAFB5*) were characterized [18]. The results suggested that *PslAFB5* is more involved in flowering and early fruit development processes with minor contribution during fruit maturation and ripening; however, both *PslTIR1* and *PslAFB2* proteins play important roles in mediating overall reproductive growth development. We provided a set of evidence that the three proteins are components of an SCF ubiquitin–ligase complex. They are able to assemble co-receptor complexes with different Aux/IAAs that play distinct roles in mediating auxin responses. To gain a broader insight into their potential role in plant growth and fruit development, the auxin receptor *PslTIR1* was selected to evaluate the physiological and molecular consequences due to overexpression in tomato.

### Overexpression of PslTIR1 disturb the auxin-responsive pathway in tomato

**Fig. 1:**
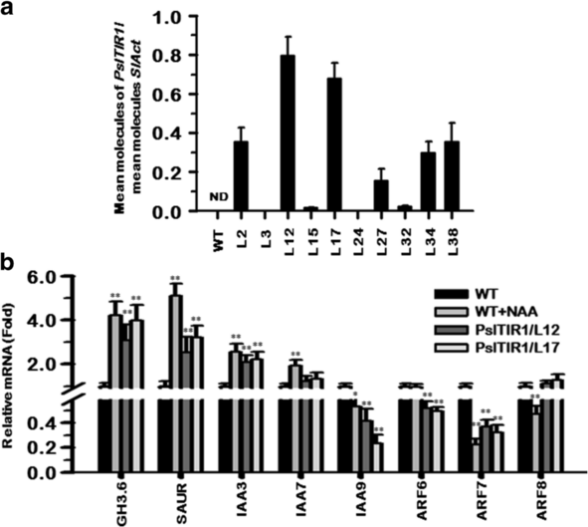
**a** *PslTIR1* transgene accumulation in 10 week-old leaf samples from WT and the different transgenic tomato events were assessed by qPCR. Standard curves were used to calculate the numbers of target gene molecules per sample, which were then normalized relative to *SlAct* expression. ND means non-detectable. **b** Transcript accumulation of selected tomato early auxin-responsive genes assessed in 8-weak-old young leaves of WT, auxin-treated WT, and two *PslTIR1*–transgenic events. The *y*-axis in each figure refers to the mean molecules of target gene per reaction/mean molecules of *SlAct*. Each value is the mean of three biological and technical replicates with the standard error indicated. Statistically significant differences from WT (control) are indicated by (*) and (**) for the probability levels (*P* < 0.05) and (*P* < 0.01), respectively

Several independent transgenic tomato events overexpressing plum auxin receptor, *PslTIR1*, were generated and tested for T-DNA insertions (Additional file 1: Figure S1). Different levels of the *PslTIR1* transgene expression were detected in all lines tested (Fig. 1a); however, only two independent biological representatives (L12 and L17) were selected for further molecular and phenotypic characterization. To investigate the impact of *PslTIR1*–overexpression on disturbing the auxin-signalling pathway, transcript accumulation for a number of early auxin-responsive genes was assessed in WT, auxin-treated WT, and transgenic (L12 and L17) tomato seedlings (Fig. 1b). Relative to WT, transcripts for *GH3.6*, *SAUR*, and *IAA3* were increased markedly in *PslTIR1* as well as auxin-treated WT plants. *GH3* genes encode IAA-amido synthetases, which converts free auxin to its conjugated form and maintains auxin homeostasis inside a cell. As in *SlARF7*-silenced plants [14], the up-regulation of *GH3.6* indicated that its induction may compensate for excessive auxin-response in *PslTIR1*–plants. Although *IAA9* and *ARF7* dramatically declined in all plants relative to WT, a slight difference in their accumulation pattern was detected. *PslTIR1*–seedlings showed more effectiveness in suppressing *IAA9* mRNA (~70 %) than those of auxin-treated WT (~45 %). Contrary, *ARF7* down-regulation was more in auxin-treated WT (80 %) than in *PslTIR1*–seedlings (~60 %). More pronounced differences between *PslTIR1*–seedlings and auxin-treated WT were detected by analyzing *ARF6* and *ARF8* accumulation pattern. *PslTIR1*–seedlings exhibited significant reduction in *ARF6* mRNA; however, its transcription did not respond to the auxin presence in treated WT. In contrast, no significant differences of *ARF8* levels were observed in *PslTIR1*–seedlings, although auxin-treated WT showed considerably declined levels. Finally, the transcription of all tomato *F*-box/auxin receptors (*TIR1*, *AFB4*, and *AFB6*) was unchanged from the WT (data not shown). The differential capacities between auxin-treated WT and *PslTIR1*–seedlings in changing the transcription profile of the different auxin-signalling components highlighted the potential selective contribution of *PslTIR1* in mediating different aspects of plant development, which can distinguish between the dynamics of auxin responses due to increasing auxin levels and that caused by activating particular auxin-signalling pathway.

### PslTIR1–overexpression lines exhibit compact stature and altered leaf morphology

**Fig. 2:**
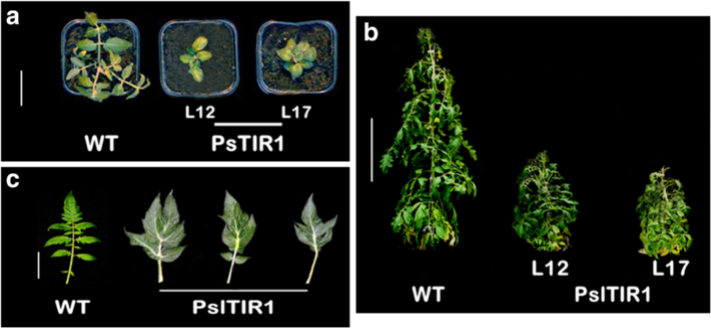
Effect of *PslTIR1*-overexpression on vegetative growth. Phenotype representative of WT and *PslTIR1*–lines (L12 and L17) at two- (**a**) and sixteen-week old (**b**), respectively, (Bars = 5 cm and 0.3 m, respectively). **c** Changes in leaf morphology and structure due to *PslTIR1*–overexpression (Bar = 2.5 cm)

Overexpression of *PslTIR1* led to a wide range of disturbances in general growth and development consistent with altered auxin-responsiveness. From early plant development, transgenic seedlings exhibited thicker stems and shorter internode (Fig. 2a). This altered growth continued through plant life-cycle, producing a typical dwarf phenotype with ~40 % reduction in the height of adult *PslTIR1*–plants (Fig. 2b). Interestingly, a similar phenotype was observed in transgenic tomato plants overexpressing *SlTIR1* [43]. In contrast, exogenous auxin application or activating auxin-responsiveness in tomato via suppressing *SlIAA9* enhanced stem elongation and produced taller plants [16, 52]. To determine the nature of this compact phenotype, we assessed the accumulation of several tomato genes and transcription factors that have been shown to contribute to plant stature with potential regulation by auxin in WT and *PslTIR1*–plants, including tomato *DELLA*, *GA2ox*, *GA20ox*, *GA3ox*, *TIR1*, *AFB5* and *AFB6*. However, no significant differences in their accumulation pattern were detected (data not shown), suggesting a possible autonomous role played by *PslTIR1* in controlling plant stature.

Moreover, one of the most readily visible phenotype was related to leaf morphology. WT tomato leaves are unipinnately compound with a terminal leaflet and three pairs of lobed major lateral leaflets with pinnate venation. In contrast, *PslTIR1*–plants displayed significant reduction in leaf compounds. They exhibited occasional lobed simple leaves architecture with fewer pairs of lateral leaflets merged with the terminal leaflet. However, the secondary small leaflets frequently seen between the major leaflets were totally absent (Fig. 2c). Auxin is the driving force of leaf growth and pinna determination [53, 54]. Therefore, any alteration in auxin distribution or response pathways might be responsible for the changes in leaf morphology. The consistency of producing simplified leaf architecture phenotype due to *TIR1*–overexpression from two different plant species, plum and tomato, in two different tomato genetic backgrounds, *Ailsa Craig* and *MicroTom*, suggests the definite contribution of *TIR1* in patterning leaf morphogenesis and dissection ([43]; this study). So far, the tomato *ENTIRE* gene, also called *IAA9*, is the only auxin-signalling component that distinctly has been shown to mediate compound-leaf patterning via modulating auxin response [16, 49, 55, 56]. Down-regulation of *IAA9* in tomato reduced the complexity of leaf morphology, similar to that of *PslTIR1*–leaves. Several lines of evidence, including i) the capacity of PslTIR1 to form co-receptor complex with tomato IAA9 [18], ii) the dramatic suppression of *IAA9* mRNA in *PslTIR1*–seedlings, iii) the analogous phenotype of *PslTIR1*– and *SlTIR1*–leaves morphology with those of auxin-treated tomato WT plants as well as tomato *entire* and *IAA9* mutants [16, 43, 56] confirmed that TIR1 proteins might regulate the auxin-dependent leaf simplification via targeting the auxin repressor IAA9, leading to destabilization of IAA9::ARF inhibitory complex.

### PslTIR1–overexpression causes alteration in reproductive growth behavior

**Fig. 3:**
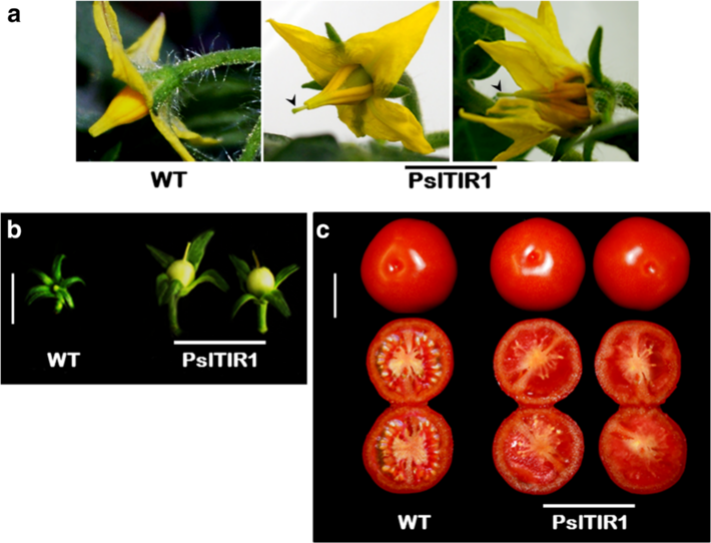
Close-up views of WT and *PslTIR1* **a** flowers at anthesis; the arrows indicate the protruding stigma. **b** Parthenocarpic fruit-set at early immature stage after emasculation; the stamen cones were removed when the flowers had not yet opened, but are ready to turn yellow (Bar = 50 mm). **c** Adult mature fruit from WT after fertilization and parthenocarpic *PslTIR1*–fruit (Bar = 6 cm)

WT tomato exhibits a typical coordinated fruit-set and development following pollination and fertilization [11]. *PslTIR1*–overexpression resulted in dramatic changes in overall reproductive growth, including flower and fruit. All transgenic plants exhibited significant reduction in the emergence of flower buds and fertility. WT produced an average of 29 ± 5 flowers/plant; however, *PslTIR1*–plants produced only 5 ± 2 flower/plant. *PslTIR1*–plants did not differ from the WT in terms of flowering time, flower size, and overall fruit development. Nonetheless, visible changes were observed in flower structure such as protruding stigma (protrudes well above the staminal cone), limiting self-pollination (Fig. 3a). The structure of open immature fused staminal cone in older flowers supported the impact of stimulated ovary growth in producing this phenotype. Our results suggested that *PslTIR1*–overexpression caused precocious fruit-set prior to anthesis independent of pollination and fertilization, which triggered the parthenocarpic fruit development. Fruit-set did not occur in emasculated WT flowers and the unfertilized flowers abscised within 3-4 d. Emasculated *PslTIR1*–flowers, by contrast, remained attached to the plant and developed into seedless fruit (Fig. 3b, c), which confirmed parthenocarpy. Despite this parthenocarpic character, *PslTIR1*–fruit were similar in appearance to WT in terms of size and skin color.

These results clearly indicated that *PslTIR1*–overexpression caused considerable disturbance in several auxin-responsive genes, resulting in alteration on typical fruit-set program and strong tendency to develop parthenocarpic fruit. In tomato, parthenocarpy fruit-set can be induced by auxin application or by modifying auxin-signalling [14, 16, 41, 42, 57]. However, several lines of evidence suggest that this phenotype is mainly due pleiotropic effect of IAA9 suppression caused by *PslTIR1*–overexpression. Compared with WT, the basal transcript levels of *IAA9* and *ARF7* were decreased in *PslTIR1*–lines, which agrees with previous studies reported the involvement of *IAA9*- and *ARF7*-suppression in parthenocarpic fruit-set [14, 16, 43]. Apparently the disturbance in the gene network involved in fruit-set either by suppression (e.g. IAA9 and ARF7) or activation (e.g. TIR1) led finally to produce similar changes in fruit-set process. Moreover, the abundance of auxin-induced *GH3.6*, *SAUR*, and *IAA3* transcripts in *PslTIR1*–lines is consistent with their accumulation profile in auxin-hypersensitive tomato mutants [14, 16, 43, 58]. Thus, it is possible to speculate that PslTIR1 positively regulate auxin-responses and fruit-set via mediating the degradation of Aux/IAA proteins, particularly IAA9.

### Effect of PslTIR1–overexpression in fruit ripening

**Fig. 4:**
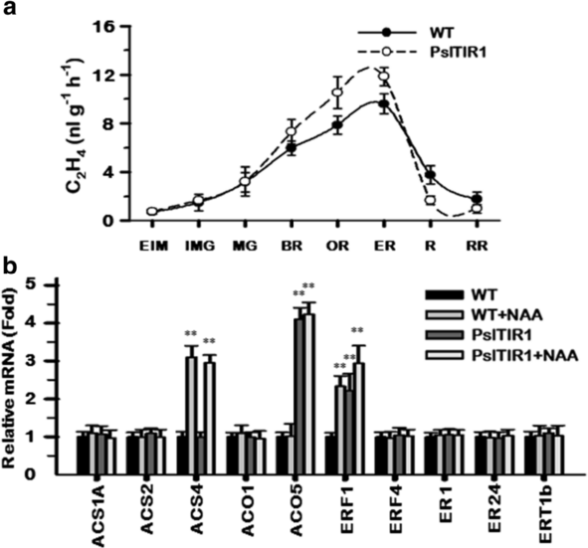
**a** Changes in ethylene production during WT and *PslTIR1*–fruit development; early-immature green (EIM), immature green (IMG), mature green (MG), breaker (BR), orange (OR), early red (ER), red (R), red-ripe (RR). **b** Transcript accumulation of selected tomato genes involved in defining ethylene production levels and fruit ripening was assessed in red WT and *PslTIR1*–fruit treated or not with auxin. The *y*-axis in (**a**) represents the changes in ethylene levels (nl g^-1^ h^-1^). Statistically significant differences from WT (control) are indicated by (**) for the probability levels (*P* < 0.01). Other details as in Fig. 1

In climacteric fruits, auxin can enhance ripening and ethylene production, acting at least partially by triggering the transcription of several ethylene biosynthesis and signalling component elements [6, 13, 32, 59]. To examine the contribution of *PslTIR1*–overexpression, we monitored the ethylene production from early immature green until red-ripe stages in WT and transgenic fruits. All fruits exhibited progressive ethylene production during ripening with no significant differences between WT and transgenic fruits (Fig. 4a). To confirm this, the accumulation profile of a set of genes that are actively involved in ethylene production and fruit ripening was assessed in red WT and transgenic fruit with or without auxin treatment, using qPCR (Fig. 4b, Additional file 1: Table S1). Analysis of expression data indicated that the accumulation profile of the different ethylene- and ripening-related transcripts in *PslTIR1*–fruit remained identical to that in the WT and did not visibly respond to auxin treatment, excluding those of *ACS4*, *ACO5* and *ERF1*. The accumulation profile of *ACS4* indicated that its transcription was triggered by auxin, but *PslTIR1* is not involved in this stimulatory effect. Although *ACO5* is dramatically increased in *PslTIR1*–fruit, its response to auxin treatment suggested the auxin-independent accumulation pattern. Interestingly, considerable high levels of *ACO5* were found to be associated with tomato parthenocarpic fruit development [60]. Thus, the accumulation of *ACO5* in *PslTIR1*–fruit seemed to be parthenocarpic-dependent rather than auxin-dependent. *ERF1* showed a typical auxin-dependence accumulation in terms of response to auxin application and *PslTIR1*–dependent regulation. These results suggested that *PslTIR1*–overexpression is not involved in the crosstalk regulatory mechanism between ethylene and auxin signalling.

### Effect of PslTIR1–overexpression in fruit shelf-life trait

**Fig. 5:**
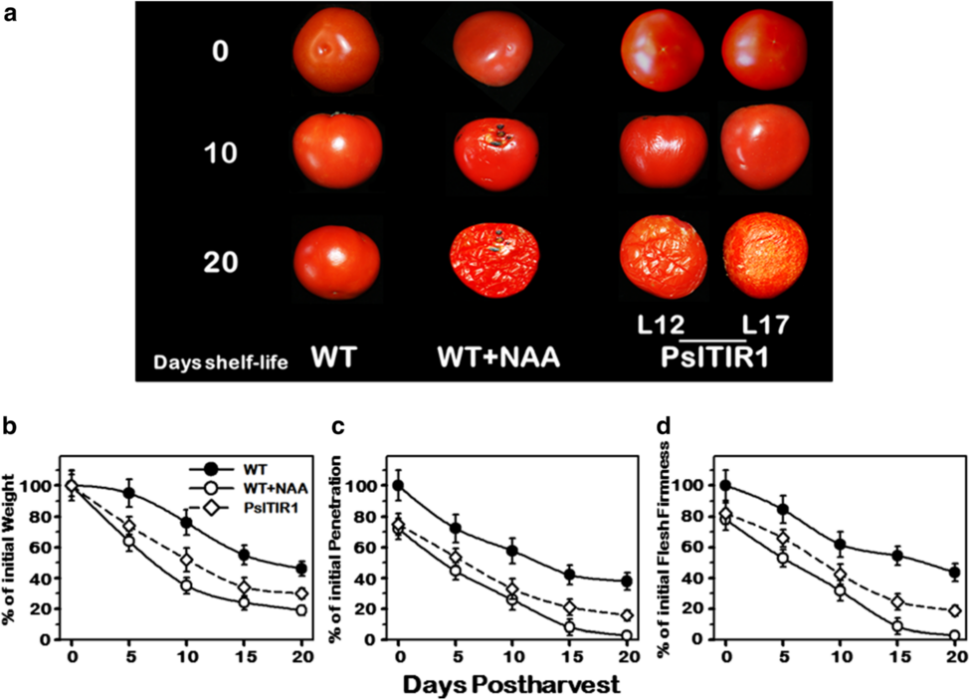
Overexpression of *PslTIR1* alters tomato fruit shelf-life characteristics. **a** Transgenic (L12, L17), auxin-treated WT and untreated WT (control) fruits were stored at room temperature (23 °C and 60 % relative humidity). Time after harvest is specified by days. Shelf-life fruit characteristics were determined as a % of initial weight loss (**b**), penetration loss (**c**), and firmness compression loss (**d**) during shelf-life storage of auxin-treated WT and transgenic fruits, comparing with WT. The values per fruit were recorded every five days until they lost their texture and structure integrity. Values represent mean ± SE (*n* = 5)

Ethylene and its biosynthetic genes are involved in the regulation of fruit softening and maintenance of shelf-life in several fleshy fruits [61–63]. Our results suggested the minor contribution of *PslTIR1* in mediating autocatalytic ethylene production and in coordinating tomato fruit ripening. This prompted us to assess the postharvest behavior of *PslTIR1*–fruit to determine their shelf-life capacity. Texture of fleshy fruit not only affects consumer preference, but also has a significant impact on shelf-life and storability. WT and transgenic fruits were harvested at early breaker stage and stored at room temperature until they reached complete deterioration (~20 d after storage). To confirm any potential role of auxin, shelf-life characteristics were also assessed in WT fruit treated with auxin. *PslTIR1* and auxin-treated WT fruits broke down faster than WT with much more and rapid deterioration in auxin-treated WT fruit (Fig. 5a).

The shelf-life reduction of *PslTIR1*–fruit had driven us to assess several shelf-life parameters to better evaluate the impact of auxin. The shelf-life was measured mainly by weight loss, penetration strength, and firmness during storage of fruits. Initially, no significant changes in weight loss were observed. However, as ripening proceeded, the weight loss significantly increased in *PslTIR1*–fruit relative to control (Fig. 5b), resulting in 70 % ± 5.3 loss of weight by the end of storage period; while WT fruit exhibited only 54 % ± 4.7 weight loss. Interestingly, treatment of WT with auxin increased the rate of weight loss, even higher than *PslTIR1*–fruit (81 % ± 6.6 weight loss). During storage, fruit firmness data represented by skin mechanical strength and flesh compression, showed that control WT fruit were substantially firmer than that of *PslTIR1* and auxin-treated tomatoes (Fig. 5c, d). By the end of storage duration, *PslTIR1* and auxin-treated fruits were 47 % and 61 % less in penetration mass, and 59 and 72 % less in flesh firmness than control, respectively. Comparing with *PslTIR1*–fruit, the stronger effect of auxin treatment in fruit shelf-life characteristics suggested that PslTIR1 is not the only auxin-related protein involved in mediating fruit shelf-life events, particularly weight loss and firmness.

### Cell-wall metabolism genes differentially respond in PslTIR1–overexpressed tomato

The alterations in shelf-life characteristics of *PslTIR1*–fruit prompted us to investigate whether *PslTIR1*–overexpression impacts genes encoding proteins involved in cell-wall degradation. To establish the regulatory mechanism(s) of fruit softening during ripening, the expression of a set of cell-wall metabolism genes that are involved in defining tomato fruit firmness (Additional file 1: Table S1) was quantified in WT and *PslTIR1*–fruit at harvest (early breaker stage; B) and after reaching ripening red stage. Further, the transcription of the different cell-wall metabolism genes was assessed in red WT and *PslTIR1* fruits pre-exposed to several treatments that can alter auxin and ethylene signalling to determine the involvement of ethylene and auxin in fruit softening.

**Fig. 6:**
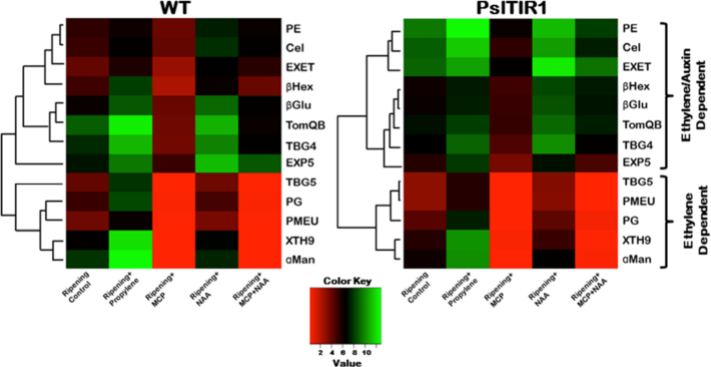
Hierarchical clustering analysis of the transcript levels of cell-wall metabolism genes. The clustering analysis was performed on genes differentially expressed between WT and *PslTIR1* fruits at ripening and in ripen fruits pre-exposed to different treatments that alter ethylene and auxin response. Green boxes indicate higher levels of expression, and red boxes indicate lower expression levels compared with the WT (Ripening Control). The color brightness is directly proportional to the expression ratio, according to the color scale at the bottom of the figure

Among the twelve cell-wall metabolism genes tested, eight transcripts including *βHex*, *TomQB*, *EXET*, *EXP5*, *TBG4*, *βGlu*, *PE* and *Cel* were initially higher in *PslTIR1*–fruit than WT at early breaker stage (Additional file 1: Figure S2). However, all the 12 transcripts dramatically increased in both WT and transgenics with the progression in fruit ripening. Analysis of expression data in red WT fruit (Ripening Control) differentiated the transcripts based on their responses to different treatments into two main groups (Fig. 6). Group-1 includes all mRNAs greatly accumulated in an ethylene-dependent manner, with no visible response to any of auxin-related treatments (*ɑMan*, *PME*, *PG*, and *XTH9*). The second group contains all transcripts up-regulated in both auxin- and ethylene-dependent manners, including *βHex*, *TomQB*, *EXET*, *EXP5*, *TBG4*, *βGlu*, *PE* and *Cel*. Although their expression levels significantly declined in MCP-treated WT fruit, they considerably accumulated in *PslTIR1*–fruit treated with MCP or in WT and *PslTIR1*–fruit treated with MCP and auxin (Additional file 1: Figure S2).

Cell-wall metabolism during ripening is an important aspect and has been explored extensively. Both ethylene-dependent and -independent softening pathways coexist to coordinate climacteric fruit ripening process [6, 26]. Given its almost ubiquitous importance, it was not surprising that auxin plays a prominent role in coordinating different aspects of fruit ripening [1]. The impact of auxin in mediating fruit firmness by regulating the fine pectin structure and tissue architecture has been previously acknowledged [31–33]. Moreover, down-regulation of tomato *APETALA2a* gene suggested that some of the ethylene-mediated responses are performed through auxin action, at least in part, during ripening [64].

## Conclusions

Plant hormones are long known to be tightly associated with fruit development and fruit ripening. Although, ethylene is considered a major player in coordinating the ripening-related events in climacteric fruit, emerging evidences highlighted auxin as another integral player in this dynamic mechanism. The present study provides another line of evidence through the overexpression of a plum auxin receptor, *PslTIR1,* in tomato. Although transgenic tomato plants showed signs of auxin-hypersensitivity, which are usually connected to the overexpression of auxin positive regulators, the accelerated softening of transgenic fruits represents a novel phenotype that links auxin directly to the ripening process. In our previous study, we found that the accumulation of *PslTIR1* mRNA is well correlated with high ethylene levels, high auxin content, and rapid loss of firmness in plum fruit [18]. In the present study we demonstrated that PslTIR1 protein is not involved in stimulating autocatalytic ethylene production associated with fruit ripening; however, it is more implicated in fruit softening events through controlling the transcription cell-wall disassembly related genes independent of ethylene action. Altogether, this study shows another strand in the molecular network that orchestrates the progression of ripening in climacteric fruit.

## Availability of supporting data

All supporting data are included as additional files.

## Additional file

Additional file 1: Table S1. The oligonucleotide primers. **Figure S1.** PCR with genomic DNA of potential positive *PslTIR1*–transgenic lines. **Figure S2.** Steady-state transcript levels of several tomato cell-wall disassembly genes. (DOCX 251 kb)

## Abbreviations

1-MCP: 1-methylcyclopropene
ACO: 1-aminocyclopropane-1-carboxylate oxidase
ACS: 1-aminocyclopropane-1-carboxylate synthase
AFB: Auxin Signaling F-Box
ɑGal: ɑ-galactosidase
ɑMan: ɑ-mannosidase
ARF: auxin response factor
Aux/IAA: auxin-signalling repressors
Cel: endo-1,4-β-glucanase
DELLA: gibberellin-signalling repressors
ERF: ethylene response factor
EXET: Endo-xyloglucan transferase
Exp: expansin
GA20ox: gibberellin 20-oxidase
GA2ox: gibberellin 2-oxidase
GA3ox: gibberellin 3-oxidase
GH3: auxin-conjugating enzyme
NAA: 1-naphthalene acetic acid
PE: Pectinesterase
PG: polygalacturonase
PME: pectin methylesterase
SAUR: small aux-in-up RNAs
SCF: Skp/Cullin/F-box complex
TBG: β-galactosidase
TIR1: transport inhibitor response 1
TomQb: Glucan endo-1,3-β-D-glucosidase
WT: wild-type
XTH: xyloglucan endotransglucosylase-hydrolase
βGLU: β-1,3-glucanase
βHex: β-hexosaminidase
