# Supplementary material for: Overexpression of plum auxin receptor PslTIR1 in tomato alters plant growth, fruit development and fruit shelf-life characteristics
Source: BMC Plant Biol. 2016 Feb 29;16:56. doi: 10.1186/s12870-016-0746-z (PMC4772300; doi:10.1186/s12870-016-0746-z)

**Additional Information**

Overexpression of plum auxin receptor PslTIR1 in tomato alters numerous developmental aspects of vegetative and reproductive growth and fruit shelf-life characteristics.

El-Sharkawy I, Sherif S, El Kayal W, Jones B, Li Z, Sullivan AJ, Jayasankar S*.

**This PDF file includes:**

- Supplemental Table.

- Supplemental Figure.

| **Table S1.** The oligonucleotide primers. | | | |
| --- | --- | --- | --- |
| Name | Oligonucleotide sequence | Target ID | Target description |
| **1-PslTIR1(F)** | **5’**-ATATAACTAGTATGCTGAAAATGGCGAAC-**3’** | KJ018741 | F-box auxin receptor TIR1-Like |
| **2-PslTIR1(R)** | **5’**-ATATAGGTTACCAGTAACCCTCACTGCAGA-**3’** |  |  |
| **Tomato qPCR primers** | | | |
| **3-Act(FQ)** | **5’**-TGTCCCTATTTACGAGGGTTA-**3’** | BT013524 | β-actin |
| **4-Act(RQ)** | **5’**-CAGTTAAATCACGACCAGCAA-**3’** |  |  |
| **Auxin-associated genes** | | | |
| **5-GH3.6(FQ)** | **5’**-TGTGACATAGTCCCAGTAA-**3’** | EU543264 | GH3-like protein |
| **6-GH3.6(RQ)** | **5’**-TTGAAATGGAATGTAGTAA-**3’** |  |  |
| **7-SAUR(FQ)** | **5’**-ATGTTGGGGAAAAGCAGAAG-**3’** | XM_004230933 | auxin-induced protein 15A-like |
| **8-SAUR(RQ)** | **5’**-ACCCATCGGATGATTAAAGC-**3’** |  |  |
| **9-IAA3(FQ)** | **5’**-GATCATACAGGAAAAATCAT-**3’** | JN379433 | Aux/IAA transcription factor 3 |
| **10-IAA3(RQ)** | **5’**-TCCTTCTCTTTCTGAATACA-**3’** |  |  |
| **11-IAA7(FQ)** | **5’**-ACTCAACCTCCATCATAAT-**3’** | JN379435 | Aux/IAA transcription factor 7 |
| **12-IAA7(RQ))** | **5’**-ACCCCACCACTTGAGCCTT-**3’** |  |  |
| **13-IAA9(FQ)** | **5’**-CCCCTTGCACCCTTCCA-**3’** | JN379437 | Aux/IAA transcription factor 9 |
| **14-IAA9(RQ))** | **5’**-AGCGTCTGAAAATCCTC-**3’** |  |  |
| **15-ARF6(FQ)** | **5’**-CCAACATATCCCTAGTACTTC-**3’** | HM594684 | Auxin response factor 6 |
| **16-ARF6(RQ))** | **5’**-GTGCCTGAGATATTAGTTGGT-**3’** |  |  |
| **17-ARF7(FQ)** | **5’**-TCAACTCCTCAAACATACCT-**3’** | EF121545 | Auxin response factor 7 |
| **18-ARF7(RQ))** | **5’**-TGAACTATCCAAATAATCCA-**3’** |  |  |
| **19-ARF8(FQ)** | **5’**-TGACATCGAATGGAAATTCA-**3’** | EF667342 | Auxin response factor 8 |
| **20-ARF8(RQ))** | **5’**-GTCTCTTAGCACTAACAAAC-**3’** |  |  |
| **21-TIR1(FQ)** | **5’**-TTGCTGGTGATAGCGATCTG-**3’** | AK320427 | *F*-box auxin receptor TIR1-Like |
| **22-TIR1(RQ)** | **5’**-GAGAGCCTTGTCTCCAAACG-**3’** |  |  |
| **23-AFB4(FQ)** | **5’**-GGTTACGACCCGTTTCAAGA-**3’** | GU079663 | *F*-box auxin receptor TIR1-Like |
| **24-AFB4(RQ)** | **5’**-AAAGTGAGCACCCCAATCTG-**3’** |  |  |
| **25-AFB6(FQ)** | **5’**-GCATTCCCAGATGAGGTGTT-**3’** | AK324204 | *F*-box auxin receptor TIR1-Like |
| **26-AFB6(RQ)** | **5’**-CCAATCTTTGCACACCAATG-**3’** |  |  |
| **Ripening-associated genes** | | | |
| **27-ACS1A(FQ)** | **5’**-GGTGCTCATGAAATGCTTG-**3’** | Q9FY02 | 1-aminocyclopropane-1-carboxylate synthase 1A |
| **28-ACS1A(RQ)** | **5’**-TGGGACCAAAAAGGCATCA-**3’** |  |  |
| **29-ACS2(FQ)** | **5’**-TTCGATGGAACAGAAGCAAC-**3’** | AY326958 | 1-aminocyclopropane-1-carboxylate synthase 2 |
| **30-ACS2(RQ)** | **5’**-GGGAGGAATAGGTGACGAAA-**3’** |  |  |
| **31-ACS4(FQ)** | **5’**-TTGCGGTCATTGTTGAAAGA-**3’** | M88487 | 1-aminocyclopropane-1-carboxylate synthase 4 |
| **32-ACS4(RQ)** | **5’**-CCTACCTCCGAGCAATTGAA-**3’** |  |  |

| **Continue Table S1.** The oligonucleotide primers. | | | |
| --- | --- | --- | --- |
| Name | Oligonucleotide sequence | Target ID | Target description |
| **Ripening-associated genes** | | | |
| **33-ACO1(FQ)** | **5’**-TGCGCCATCTTCCTACTTCT-**3’** | NM_001247095 | 1-aminocyclopropane-1-carboxylate oxidase 1 |
| **34-ACO1(RQ)** | **5’**-CCTTGATCAAATCGGGCTTA-**3’** |  |  |
| **35-ACO5(FQ)** | **5’**-AGAATGGAAACATCAGCAA-**3’** | Q6A1K7 | 1-aminocyclopropane-1-carboxylate oxidase 5 |
| **36-ACO5(RQ)** | **5’**-GCTGGACGATGCCAAACAA-**3’** |  |  |
| **37-ERF1(FQ)** | **5’**-GTGTAGCGGCTAAGCAAGC-**3’** | Q84XB3 | Ethylene response factor 1 |
| **38-ERF1(RQ)** | **5’**-TCCATTTGGCAACCAACTT-**3’** |  |  |
| **39-ERF4(FQ)** | **5’**-CAATTCCTCATACCCAAATGA-**3’** | Q84XB0 | Ethylene response factor 4 |
| **40-ERF4(RQ)** | **5’**-TCGGAGCGGAGATTGAAGTAA-**3’** |  |  |
| **41-ER1(FQ)** | **5’**-GGAGGCAAATAAGTCTATGGT-**3’** | P20076 | Fruit-ripening protein |
| **42-ER1(RQ)** | **5’**-GAGAGATTGAAAGCATGATGA-**3’** |  |  |
| **43-ER24(FQ)** | **5’**-AGGCCCAAGACCTGAAGGA-**3’** | Q9SWW1 | Ethylene-responsive transcriptional coactivator |
| **44-ER24(RQ)** | **5’**-TTGTGCTCCAGCTCGCAAT-**3’** |  |  |
| **45-ERT1b(FQ)** | **5’**-AACCGTTCTTGTGGGTTATT-**3’** | ×72729.1 | Ripening-related mRNA |
| **46-ERT1b(RQ)** | **5’**-TGCCTCAGCACATTGAAAGC-**3’** |  |  |
| **Cell-wall metabolism-associated genes** | | | |
| **47-ɑMan(FQ)** | **5’**-GTTGCTGCTTCAATACCACA-**3’** | EU244853 | ɑ-mannosidase |
| **48-ɑMan(RQ)** | **5’**-CTCCAAAGAGCTTCTAACCTG-**3’** |  |  |
| **49-βHex(FQ)** | **5’**-TATGTTCTGGTGGCCCG-**3’** | EU244854 | β-hexosaminidase |
| **50-βHex(RQ)** | **5’**-CTGCTCCTCCGTGAAAG-**3’** |  |  |
| **51-PME(FQ)** | **5’**-GACGGAAGCGGAGATTACA-**3’** | Q43143 | Pectin methylesterase |
| **52-PME(RQ)** | **5’**-TCTTCTCTGGCGCTTTTCGT-**3’** |  |  |
| **53-EXET(FQ)** | **5’**-TATCCTAGAAGGCCAGTAGAT-**3’** | Q40144 | Endo-xyloglucan transferase |
| **54-EXET(RQ)** | **5’**-GACTAGCCCAACTTGGCTCAT-**3’** |  |  |
| **55-TomQb(FQ)** | **5’**-CGTGCAAATCCTGACCTTCA-**3’** | Q42890 | Glucan endo-1,3-β-D-glucosidase |
| **56-TomQb(RQ)** | **5’**-GACCCAACGATTCGCGTTAG-**3’** |  |  |
| **57-Exp5(FQ)** | **5’**-ATCAATGCACATGCCACTTTTT-**3’** | Q9ZP31 | Expansin |
| **58-Exp5(RQ)** | **5’**-GCACCACCCATAGTTCCAGAA-**3’** |  |  |
| **59-PG(FQ)** | **5’**-GCCTTGTAAGTCAGCCAAT-**3’** | Q9ZPI8 | Polygalacturonase |
| **60-PG(RQ)** | **5’**-TCCATGCCACTGGGTCCTT-**3’** |  |  |
| **61-TBG4(FQ)** | **5’**-GATTGTCTTGGCAGTCATAC-**3’** | AF02039 | β-galactosidase |
| **62-TBG4(RQ)** | **5’**-TACCAGCTCTTAACTTCACG-**3’** |  |  |
| **63-TBG5(FQ)** | **5’**-GCGACTGTGGGACTTCAGAA-**3’** | Q9LLT0 | β-galactosidase |
| **64-TBG5(RQ)** | **5’**-CGTAATCCCTGCTCCCTTGA-**3’** |  |  |
| **65-βGLU(FQ)** | **5’**-TCGCCACCAACATTCACATAA-**3’** | Q01413 | β-1,3-glucanase |
| **66-βGLU(RQ)** | **5’**-TTCCCCATCATTCCATAACAA-**3’** |  |  |
| **67-PE(FQ)** | **5’**-TACCGCCAGGGAGAAATAAC-**3’** | Z94058 | Pectinesterase |
| **68-PE(RQ)** | **5’**-GCTTTGTGGAATGCAAGAGA-**3’** |  |  |
| **69-XTH9(FQ)** | **5’**-TCTGGAATCCTCATCGCATA-**3’** | NM_001247549 | Xyloglucan endotransglucosylase-hydrolase |
| **70-XTH9(RQ)** | **5’**-TGCTTGACTCTTGGGAAATG-**3’** |  |  |
| **71-ɑGal(FQ)** | **5’**-GACTCGCAGGGAAATATGGT-**3’** | AF191823 | ɑ-galactosidase |
| **72-ɑGal(RQ)** | **5’**-TTTTTGCGTCTTGTTCTTCG-**3’** |  |  |
| **73-Cel3(FQ)** | **5’**-CAGCTGGGATAACAAGCTCA-**3’** | NM_001247014 | Endo-1,4-β-glucanase |
| **74-Cel3(RQ)** | **5’**-GGATATCCAGGGCTCAGAAA-**3’** |  |  |

**Fig. S1.** PCR with genomic DNA of potential positive *PslTIR1*–transgenic lines.
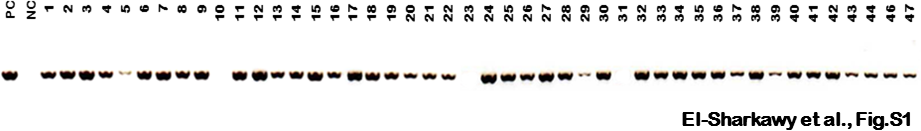
PC and NC in first and second lane refers to positive and negative control, respectively.

**Fig. S2.** Steady-state transcript levels of several tomato cell-wall disassembly genes. Transgenic and WT fruits were harvested at early breaker stage, treated with propylene (R+E), 1-MCP (R+M), auxin-NAA (R+N), and mutual treatment of MCP and auxin (R+M+N). Non-treated fruit were used as control. All fruit were stored at room temperature (23°C and 60% relative humidity) until reaching red stage. Results represent data from three biological and three technical replicates. The *y*-axis refers to the fold change in the target gene levels relative to its levels in fruit at harvest. Statistically significant differences between untreated ripe fruit (R) and ripe fruit from different treatments are indicated by (*) and (**) for the probability levels (P<0.05) and (P<0.01), respectively. Other details as in Fig. 1
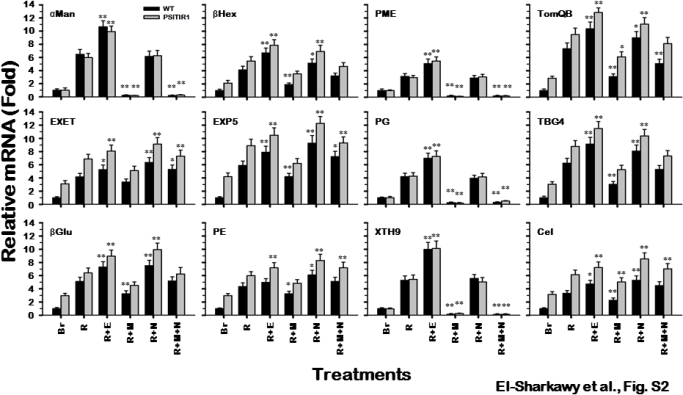

Supplement: Additional file 1: Table S1. — The oligonucleotide primers. Figure S1. PCR with genomic DNA of potential positive PslTIR1–transgenic lines. Figure S2. Steady-state transcript levels of several tomato cell-wall disassembly genes. (DOCX 251 kb) [file 12870_2016_746_MOESM1_ESM.docx]
